# The complete chloroplast genome of *Agave amaniensis* (Asparagales: Asparagaceae: Agavoideae)

**DOI:** 10.1080/23802359.2022.2109440

**Published:** 2022-08-22

**Authors:** Bochao Xu, Shibei Tan, Xu Qin, Xing Huang, Jingen Xi, Helong Chen, Jianfeng Qin, Tao Chen, Kexian Yi

**Affiliations:** a School of Life Sciences, Hainan University, Haikou, PR China; b Environment and Plant Protection Institute, Chinese Academy of Tropical Agricultural Sciences, Haikou, PR China; c Guangxi Subtropical Crops Research Institute, Nanning, PR China; d Ministry of Agriculture and Rural Affairs, Key Laboratory of Integrated Pest Management on Tropical Crops, Haikou, PR China; e Hainan Key Laboratory for Monitoring and Control of Tropical Agricultural Pests, Haikou, PR China

**Keywords:** *Agave amaniensis*, chloroplast genome, phylogenetic tree

## Abstract

*Agave amaniensis* Trel. & W. Nowell (1933) has long been used for phytosteroid production, which is also one of the parents of the famous *Agave* hybrid cultivar 11648 for sisal fiber production. However, its systematic position and phylogenetic relationship remains unknown at the chloroplast (cp) genome level. Therefore, we have sequenced and assembled the cp genome of *A. amaniensis* via Illumina sequencing. The cp genome is 157,282 bp in length with a GC content of 37.84%. A large single-copy region of 85,899 bp, a small single-copy region of 18,233 bp, and inverted repeat regions of 26,575 bp were found in the cp genome. Based on the annotation, 86 protein-coding genes, eight rRNAs, and 38 tRNAs were identified in the cp genome with total lengths of 78,981 bp, 9050 bp, and 2867 bp, respectively. The phylogenetic tree indicates that *A. amaniensis* is closely related with *A*. H11648, *A. angustifolia*, and *A. americana*.

## Background

Agave plants are widely cultivated in the tropical areas of the world for food, beverage, fiber, and medicine production (Huang et al. 2019). Among the 166 cultivated *Agave* species, *Agave amaniensis* Trel. & W. Nowell (1933) has long been used for phytosteroid production (Indrayanto et al. 1993; Gil-Vega et al. 2006). This species also serves as one of the parents of the famous *Agave* hybrid cultivar 11648, which is cultivated for sisal fiber production worldwide (Huang et al. 2018). However, the systematic position and phylogenetic relationship of *A. amaniensis* remains unknown at the chloroplast (cp) genome level. Therefore, we have sequenced and assembled the cp genome of *A. amaniensis* via Illumina sequencing to facilitate future studies on *Agave* cps.

## Methods

The leaves of *A. amaniensis* were collected from a two-year-old plant grown in the germplasm garden (22.90°N, 108.33°E) of the Guangxi Subtropical Crops Research Institute, Nanning, China. Several leaves were processed as specimens and stored in the Herbarium of Environment and Plant Protection Institute, Chinese Academy of Tropical Agricultural Sciences (voucher no. EPPI-jm2020012, https://eppi.catas.cn/, Xing Huang, hxalong@gmail.com). Further, DNA was extracted from the remaining leaves using the modified CTAB method and stored at −80 °C until submission to Biozeron Biotech (Shanghai, China) for sequencing (Doyle and Doyle 1987). Paired-end sequencing was performed using Illumina HiSeq 2500 (San Diego, CA). The raw data obtained were used for cp genome assembly using the NOVOPlasty software, followed by gap-filling using GapCloser (Luo et al. 2012; Dierckxsens et al. 2017). The complete cp genome was annotated using GeSeq and CPGAVAS2 (Tillich et al. 2017; Shi et al. 2019). Thereafter, the nucleotide sequences of protein-coding genes were extracted from the cp genome sequence. The merged protein-coding sequence was further aligned with those of other species using the MAFFT software (Katoh and Standley 2013). Sequence alignment was imported into the MEGA7 software to construct a maximum likelihood phylogenetic tree with 1000 bootstrap replicates (Kumar et al. 2016).

## Results

In total, 7.2 Gb of raw data were generated from Illumina sequencing, which were deposited to the SRA database (accession no. PRJNA705737). The assembled cp genome of *A. amaniensis* was submitted to the GenBank database (accession no. MW679302). The cp genome is 157,282 bp in length with a GC content of 37.84%. A large single-copy region of 85,899 bp, a small single-copy region of 18,233 bp, and inverted repeat regions of 26,575 bp were found. Based on the genome annotation, 86 protein-coding genes, eight rRNAs, and 38 tRNAs were identified in the cp genome with total lengths of 78,981 bp, 9050 bp, and 2867 bp, respectively.

**Figure 1.**
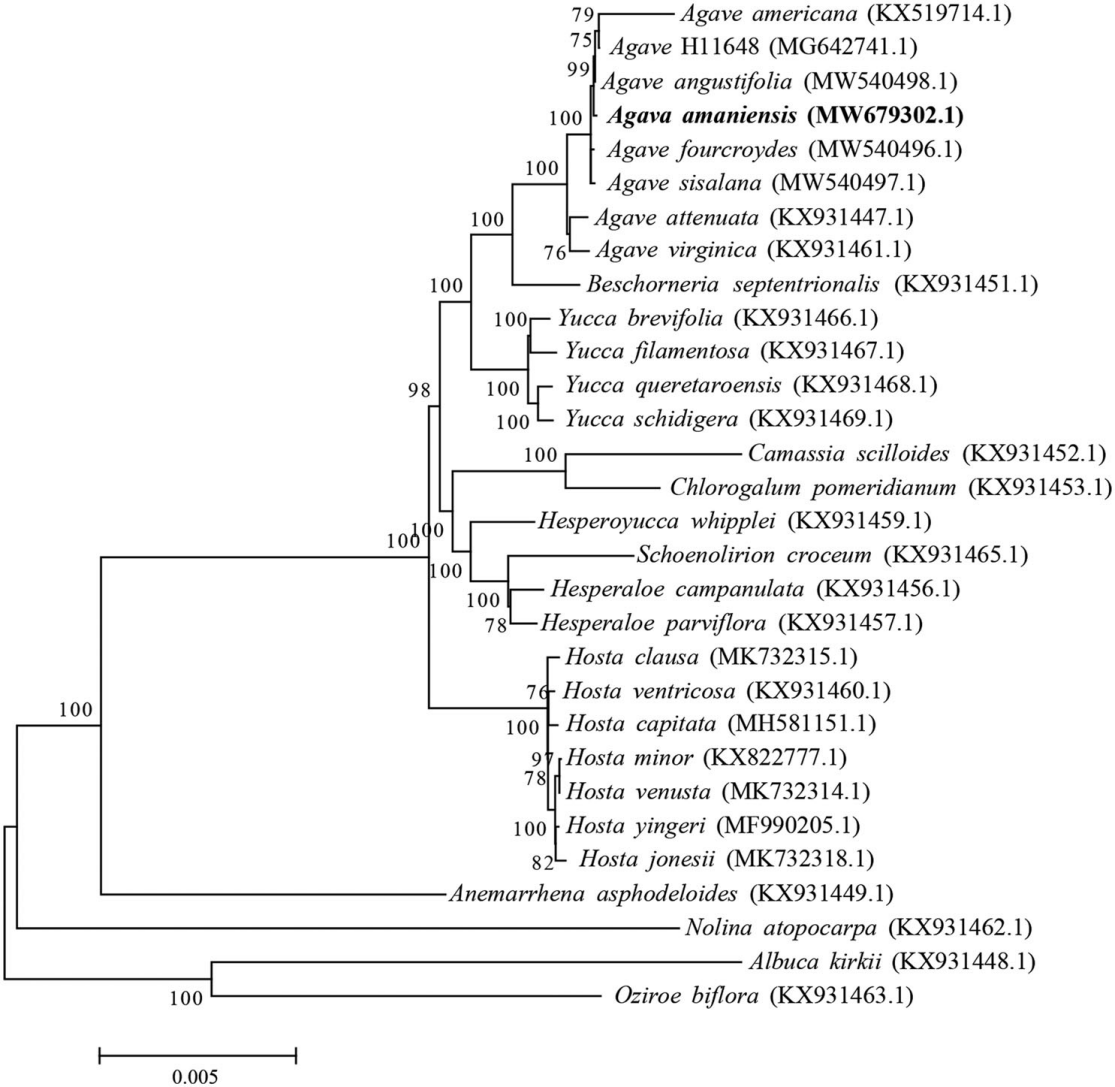
The maximum-likelihood phylogenetic tree of 30 species, including 27 Agavoideae species and three other species (*Albuca kirkii*, *Nolina atopocarpa*, and *Oziroe biflora*) as outgroup. The nucleotide sequences of protein-coding genes were extracted and merged from each cp genome for sequence alignment.

The cp genome sequences of 30 species, 27 Agavoideae species, and three other species (*Albuca kirkii*, *Nolina atopocarpa*, and *Oziroe biflora*) as outgroup were utilized for the construction of the phylogenetic tree (Qin et al. 2021). The results indicate that *A. amaniensis* is closely related with *A*. H11648, *A. angustifolia*, and *A. americana* (Figure 1).

## Data Availability

The genome sequence data that support the findings of this study are openly available in GenBank of NCBI at https://www.ncbi.nlm.nih.gov/nuccore/ under the accession MW679302. The accession numbers of BioProject, SRA, and Bio-Sample are PRJNA705737, SRS8376943, and SAMN18100000, respectively.
